# Geometric and biomechanical modeling aided by machine learning improves the prediction of growth and rupture of small abdominal aortic aneurysms

**DOI:** 10.1038/s41598-021-96512-3

**Published:** 2021-09-10

**Authors:** Moritz Lindquist Liljeqvist, Marko Bogdanovic, Antti Siika, T. Christian Gasser, Rebecka Hultgren, Joy Roy

**Affiliations:** 1 Department of Molecular Medicine and Surgery, Karolinska Institutet, Stockholm, Sweden; 2 Department of Engineering Mechanics, Royal Institute of Technology, Stockholm, Sweden; 3 Department of Vascular Surgery, Karolinska University Hospital, Stockholm, Sweden

**Keywords:** Aneurysm, Aortic diseases, Biomedical engineering

## Abstract

It remains difficult to predict when which patients with abdominal aortic aneurysm (AAA) will require surgery. The aim was to study the accuracy of geometric and biomechanical analysis of small AAAs to predict reaching the threshold for surgery, diameter growth rate and rupture or symptomatic aneurysm. 189 patients with AAAs of diameters 40–50 mm were included, 161 had undergone two CTAs. Geometric and biomechanical variables were used in prediction modelling. Classifications were evaluated with area under receiver operating characteristic curve (AUC) and regressions with correlation between observed and predicted growth rates. Compared with the baseline clinical diameter, geometric-biomechanical analysis improved prediction of reaching surgical threshold within four years (AUC 0.80 vs 0.85, *p* = 0.031) and prediction of diameter growth rate (r = 0.17 vs r = 0.38, *p* = 0.0031), mainly due to the addition of semiautomatic diameter measurements. There was a trend towards increased precision of volume growth rate prediction (r = 0.37 vs r = 0.45, *p* = 0.081). Lumen diameter and biomechanical indices were the only variables that could predict future rupture or symptomatic AAA (AUCs 0.65–0.67). Enhanced precision of diameter measurements improves the prediction of reaching the surgical threshold and diameter growth rate, while lumen diameter and biomechanical analysis predicts rupture or symptomatic AAA.

## Introduction

Abdominal aortic aneurysm (AAA) is an irreversible dilatation of the abdominal aorta to a diameter of 30 mm and above^1^. The disease is asymptomatic but with the progressive expansion of the aneurysm there is an increased risk of rupture, which is fatal in the majority of cases^2,3^. Surgery is typically considered when the AAA reaches a diameter of 55 mm but a lower threshold is recommended for women due to their increased risk of rupture^1,4^. Between their diagnosis and the AAA reaching this threshold for surgery, patients undergo repeated diameter measurements with increasing frequency as the aneurysm expands, called ‘surveillance’. However, the time to surgery, growth rate and risk of rupture remain difficult to predict with precision^1,5,6^. A small share of patients with AAAs suffer from rupture during surveillance, while on the other hand, a significant share can reach large aneurysm diameters without rupturing^7–11^. If growth rate, the future indication for surgery and rupture risk could be predicted with increased precision, surveillance and surgery could be personalized, potentially creating safer and more cost-effective management algorithms.

The diameter is the most used risk marker in AAA disease and is clinically measured ‘manually’ by ultrasound or multiplanar reconstruction on computed tomography (CT), perpendicularly to the centerline. However, present guidelines do not specify exactly how diameter should be measured and alternative methods such as semiautomatic and maximally inscribed sphere diameter measurements have been proposed to increase precision^1,12,13^. Several studies have also observed that the volume of an AAA may be a more sensitive descriptor of growth than its diameter, and that the volume growth rate is easier to predict than the diameter growth rate^14–18^.

Examples of suggested alternative predictors include characteristics of the intraluminal thrombus (ILT)^5,19–22^, tracer imaging of AAA vessel wall metabolism, calcification processes and inflammation^23–25^ as well as circulating markers of extracellular matrix degradation, inflammation, coagulation and microRNAs^26^. Further, finite element analysis (FEA) has in several retrospective case-control studies been reported to predict rupture^27–33^ but also to predict growth or future surgery^34,35^. As of yet, none of these alternative markers have been included into clinical management algorithms.

The primary aim was to determine whether geometric-biomechanical models in small AAAs could improve predictions of which aneurysms would reach the threshold for surgery within four years compared with the clinically measured diameter. Secondary aims included predicting the future growth rate and the occurrence of rupture or symptomatic AAA. To this end, we applied cross-validated prediction modeling and machine learning algorithms to three-dimensional (3D) geometric and biomechanical analyses of CT examinations.

## Methods

### Patients

In total, 189 patients with AAA were identified consecutively from the vascular surgery outpatient clinic of the Karolinska University Hospital by using two separate, consecutive, search strategies; (1) to find all patients with an AAA as well as a registered CT performed between 2009 and 2013, and (2) to evaluate all patients with AAA who visited the clinic between 2012 and 2013. Inclusion criteria were an AAA with a maximal diameter of between 40 and 50 mm, measured from an index CT examination at least four years prior to the patient record review date. Exclusion criteria were symptoms or previous surgery of the abdominal aorta at the time of the index CT, as well as aneurysms related to an infection, systemic inflammatory disease or congenital connective tissue disorders.

Of the 189 patients, 171 had complete follow-up data, i.e. did not die of unrelated causes and in whom surveillance was not terminated before reaching the surgical threshold. These patients were specifically evaluated in order to test the prediction performance in patients who were followed according to clinical protocol. Growth rate calculations were performed for the 161 patients that had undergone at least two CTAs performed with an interval of between 8 months and 8 years.

The study was conducted according to the Declaration of Helsinki. Informed consent was waived due the study’s retrospective nature and the resulting risk of losing patient material in a biased manner if patients of high age or significant comorbidity were excluded due to death or being unable to give informed consent. Further, the study did not affect the care of the included patients. The study and waiver of informed consent were approved by the Swedish Ethical Review Authority.

### Outcomes and definitions

The primary outcome was reaching the surgical threshold within four years, which was defined as the aneurysm expanding to a clinical diameter of 55 mm for men and 52 mm for women, according to our clinical protocol, being surgically treated at smaller diameters or the occurrence of rupture or symptomatic AAA within four years of the index CT. An aneurysm was considered stable if it was intact and asymptomatic with a diameter below 55 mm for men and 52 mm for women after four years of follow-up. Incomplete follow-up was defined as death from causes unrelated to the abdominal aorta or terminated surveillance for other reasons before reaching surgical threshold. These patients were placed in the stable group when all patients were considered.

The secondary outcomes were not limited to four years and comprised growth rates in diameter and volume between the index CT and follow-up CT 8 months to 8 years later, as well as the occurrence of rupture or symptomatic AAA between the time of the index CT and the record review date. The combined event of rupture or symptomatic AAA were recorded from the patients’ electronic medical records (EMR) and could occur at any time between the baseline CTA and the date of data collection. The region of Stockholm has a shared EMR system and a ruptured or symptomatic AAA diagnosed and/or treated anywhere in the region would be identified in this system. The cause of death of patients who died during surveillance was queried through the same system in order to find patients with ruptures not admitted to hospital.

### Growth rate calculations

Growth rate of diameter and volume were defined as the difference between two CT examinations, normalized into annual rates. In order to account for non-linear growth over the course of a large time span in some patients, nonlinear models of diameter and volume growth were employed:

$$Growth \ rate= \left(Exp\left(12r\right)-1\right)*{M}_{baseline }\frac{mm \ or \ c{m}^{3}}{year}$$

with the logarithmic growth factor:

$$r=\frac{1}{t}*Ln \left(\frac{{M}_{follow-up }}{{M}_{baseline}}\right)$$

In this case, *M* denotes diameter or volume, *t* refers to the time between the baseline and follow-up CTA in months and Exp(•) and Ln(•) are the exponential and natural logarithmic functions of (•), respectively^16,17^. Negative growth rate was not deemed feasible and was instead considered to be 0.

### Geometric measurements and finite element analysis

Analyses of geometry and biomechanics were performed on 189 patients and 356 CTAs with the commercially available software A4clinics Research Edition (Vascops GmbH), which has been described in detail previously^28,36,37^. In short, the software allows the segmentation of a 3D model of the AAA based on CTA images in a semi-automatic manner, where the software identifies lumen, ILT and the outer contour of the vessel wall while the investigator makes manual corrections where needed. Segmentations and analyses were performed between the level of the lowest main renal artery and the aortic bifurcation. Accessory renal arteries were ignored. The AAA vessel wall was considered to be hyperelastic, isotropic and incompressible^38,39^. The wall strength was adjusted globally based on patient sex and family history as well as locally based on local ILT thickness and the ratio between local aneurysm diameter and the expected normal diameter, all based on previous ex vivo biomechanical testing^40^. The wall strength was decreased by 50% in order to account for fatigue from pulsatile loading^39^. The finished aneurysm model was loaded with the patient-specific mean arterial pressure (MAP) and FEA was performed. The maximal diameter was re-measured by the software from the semi-automatically segmented 3D model, perpendicularly to the centerline, giving a *semiautomatic diameter*. Other output variables were total *vessel volume*, *maximal luminal diameter, lumen volume, maximal ILT thickness, ILT volume, mean ILT stress, peak wall stress (PWS) and peak wall rupture index (PWRI).* Mean ILT stress is the average estimated stress in the ILT, whereas the PWS represents the maximal stress and PWRI the maximal ratio between wall stress and wall strength in the aneurysm. The AAA diameter as measured from the CTA by the radiologist was referred to as the *clinical diameter*.

### Statistical analyses and prediction models

The outcomes of reaching surgical threshold within four years and the aneurysm growth rates were predicted by use of several commonly employed machine learning models, specifically ridge regression, least absolute shrinkage and selection operator (LASSO), k-nearest neighbors, support vector machines (SVM) with linear and polynomial kernels, random forest, gradient boosting machines and artificial neural networks. The artificial neural networks were trained by use of tensorflow/keras via R^41,42^. All other models were trained and tuned by use of internal cross-validation in R package caret, which was also used to assess variable importance^43,44^. All geometric and biomechanical variables, along with sex, smoking and any diagnosis of diabetes^2^ were included as predictors. The reference models against which the geometric-biomechanical models were compared were logistic or linear regression with the baseline clinical diameter as predictor and in those predicting which patient would reach the surgical threshold within four years, patient sex was also included as a covariate. The reference for volume growth was the baseline volume.

All predictions were performed by use of tenfold cross-validation, i.e. predicting iteratively on data not used to train the models. The cross-validated predictions were themselves iterated 100 times, each with a different seed for the automatic, (pseudo)-random splitting of data. The average predictions of the 100 iterations were evaluated by receiver operating characteristic (ROC) analysis for reaching the threshold for surgery and the Pearson correlation coefficient between the predicted and the observed value for growth rate. Differences between correlation coefficients and area under ROC curves (AUCs) were tested with the Hittner et al. test by use of the package cocor and the DeLong test by use of the pROC, respectively^45–47^.

Spearman’s correlation coefficient was used in correlation plots^48^. Continuous data were summarized as median (interquartile range, IQR) and tested with the Kruskal–Wallis test whereas categorical data were summarized as number (percent) and tested with the chi-squared test, unless stated otherwise.

## Results

### Patient characteristics and measurements

**Figure 1:**
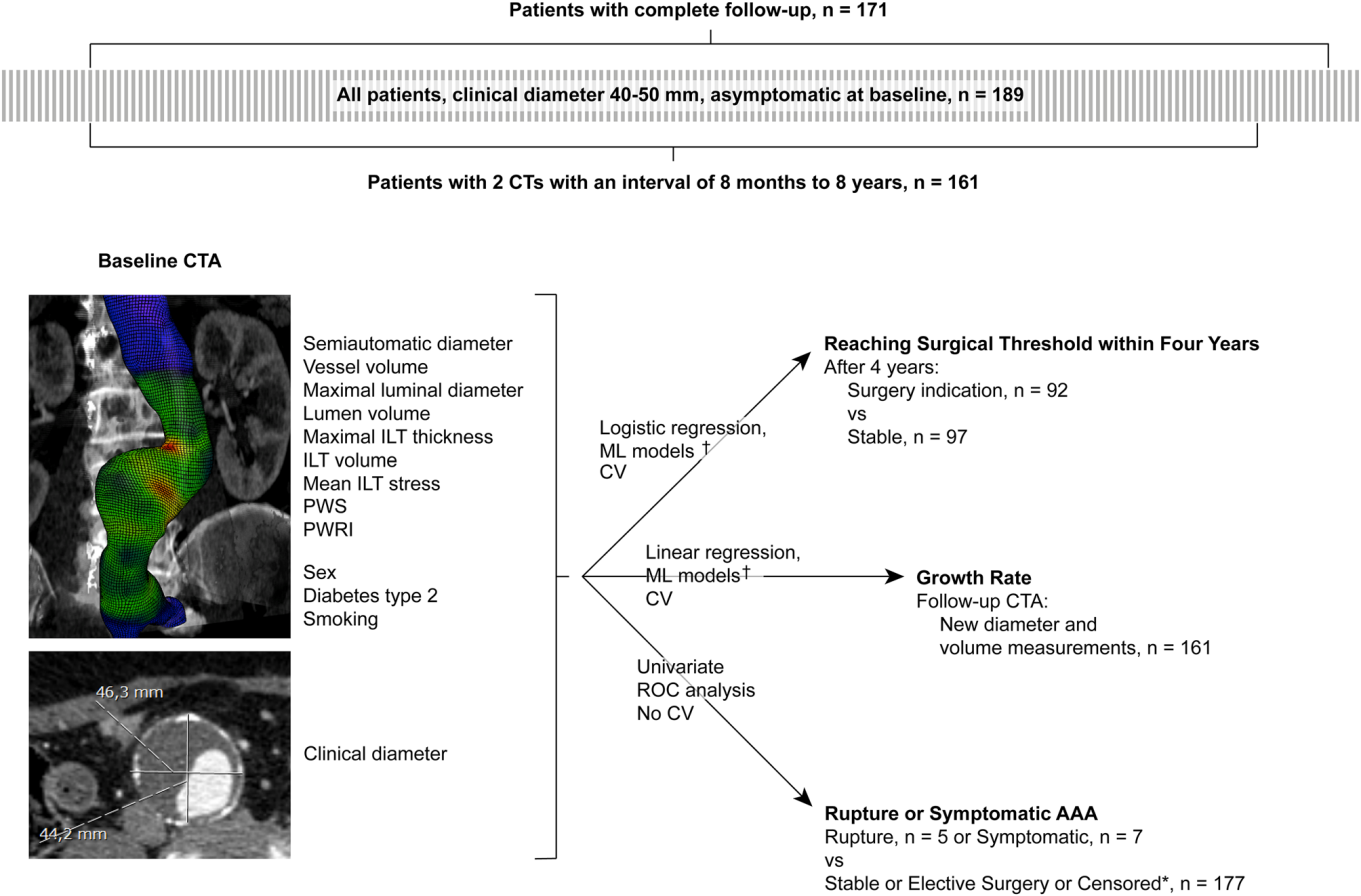
Overview of study design. *Abbreviations*; CTA: computed tomography angiography, CV: cross-validation, ILT: intraluminal thrombus, ML: machine learning, PWRI: peak wall rupture index, PWS: peak wall stress, ROC: receiver operating characteristic. *: Patients were censored if they died from unrelated causes or follow-up was terminated before surgery. †: Included prediction models were ridge regression, least absolute shrinkage and selection operator, k-nearest neighbors, support vector machines with linear and polynomial kernels, random forest, gradient boosting machines and artificial neural networks.

A total of 189 patients were included, of which 97 remained stable after four years and 92 had reached the threshold for surgery (Fig. 1). Age, smoking, family history and blood pressure at the time of the index CT did not differ between these groups (Table 1). There were, however, significant differences in patient sex as well as all geometric and biomechanical measurements. Several of these measurements correlated across all included patients (Suppl Fig. 1). The size of the ILT correlated negatively with that of the lumen. The size of the ILT also correlated negatively with PWS but not PWRI.

**Table 1:** Patient and aneurysm characteristics.

	Stable (n = 97)	Reached threshold for surgery (n = 92)	*p*
***Patient characteristics***			
**Patient sex** (female)	16 (16%)	29 (32%)	0.015
**Age**	72 (67, 77)	73 (67, 78)	0.825
**Smoking status** Current/previous/never	27 (28%)/55 (57%)/15 (15%)	33 (36%)/45 (49%)/14 (15%)	0.472
**Family History of AAA** Missing	7 (13%) 43 (44%)	16 (26%) 30 (33%)	0.084
**Type 2 diabetes**	15 (15%)	12 (13%)	0.635
**Systolic blood pressure,** mmHg Missing	140 (130, 155) 5 (5%)	140 (130, 150) 9 (10%)	0.895
**Diastolic blood pressure,** mmHg Missing	80 (80, 88) 5 (5%)	80 (79, 90) 9 10%)	0.841
***CT analysis***			
**Clinical diameter,** mm	42 (40, 45)	47 (45, 49)	< 0.001
**Semiautomatic diameter,** mm	46 (43, 48)	50 (48, 53)	< 0.001
**Max luminal diameter,** mm	34 (31, 38)	38 (33, 42)	< 0.001
**Max ILT thickness,** mm	11 (7, 15)	12 (8, 18)	0.031
**Vessel volume,** cm^3^	95 (83, 115)	122 (105, 140)	< 0.001
**Lumen volume,** cm^3^	51 (41, 65)	67 (54, 81)	< 0.001
**ILT volume,** cm^3^	24 (17, 38)	36 (20, 50)	0.008
**PWS,** kPa	172 (149, 194)	188 (167, 211)	< 0.001
**PWRI,** ratio	0.31 (0.27, 0.36)	0.37 (0.33, 0.45)	< 0.001
**Mean ILT stress,** kPa	6.4 (6.0, 7.0)	6.7 (6.1, 7.4)	0.020

Continuous and categorical data were tested with Kruskal–Wallis and chi-squared test, respectively.

*Abbreviations*: AAA; abdominal aortic aneurysm, CT; computed tomography, ILT; intraluminal thrombus, PWRI; peak wall rupture index, PWS; peak wall stress.

### Reaching surgical threshold within four years

**Figure 2:**
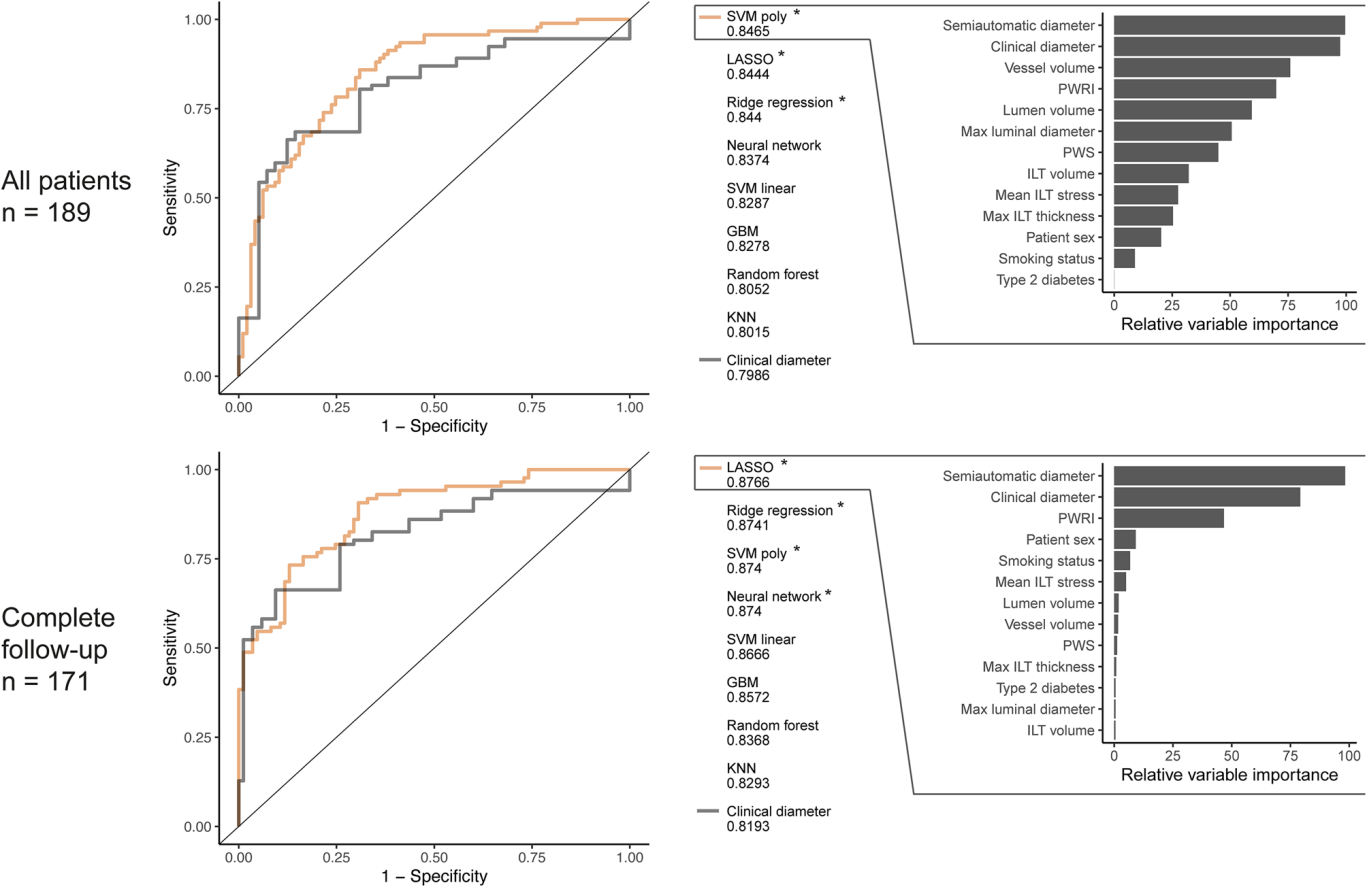
Prediction of reaching surgical threshold within four years. Predictions by different machine learning algorithms for all patients (upper row) and only those with complete follow-up (bottom row). Logistic regression of clinical diameter and patient sex served as reference. Variable importance is shown for the best model. The area under curve (AUC) is presented below each variable. Stars mark AUCs significantly larger than that of the clinical diameter reference. *Abbreviations*; GBM: gradient boosting machines, ILT: intraluminal thrombus, KNN: k-nearest neighbors, LASSO: least absolute shrinkage and selection operator, PWRI: peak wall rupture index, PWS: peak wall stress, SVM: support vector machine with linear or polynomial kernel. Notations: *: *p* < 0.05.

Geometric-biomechanical models significantly outperformed the clinical diameter and patient sex reference (Fig. 2). Specifically, the AUC for the best performing model, compared with that of the clinical diameter reference, was 0.85 vs 0.80 (*p* = 0.033). As an example, the LASSO model could achieve 100% sensitivity and 21% specificity, whereas the clinical diameter reference did not reach 100% sensitivity with any retained specificity. When patients with incomplete follow-up were excluded, so that only those followed according to clinical protocol remained (n = 171), the AUCs for best performing geometric-biomechanical model remained superior to that of the reference, 0.88 vs 0.82, *p* = 0.016, reaching 100% sensitivity and 26% specificity, whereas the reference again displayed 100% sensitivity with 0% specificity. Variable importance inspection showed that the clinical and semiautomatic diameters were the most influential features (Fig. 2). When only the clinical and semiautomatic diameters were used together as predictors, similar performance was obtained as with models including all geometric and biomechanical variables (not shown). No variable alone demonstrated significant improvement over the clinical diameter (Suppl Fig. 2).

### Growth rate of diameter and volume

**Figure 3:**
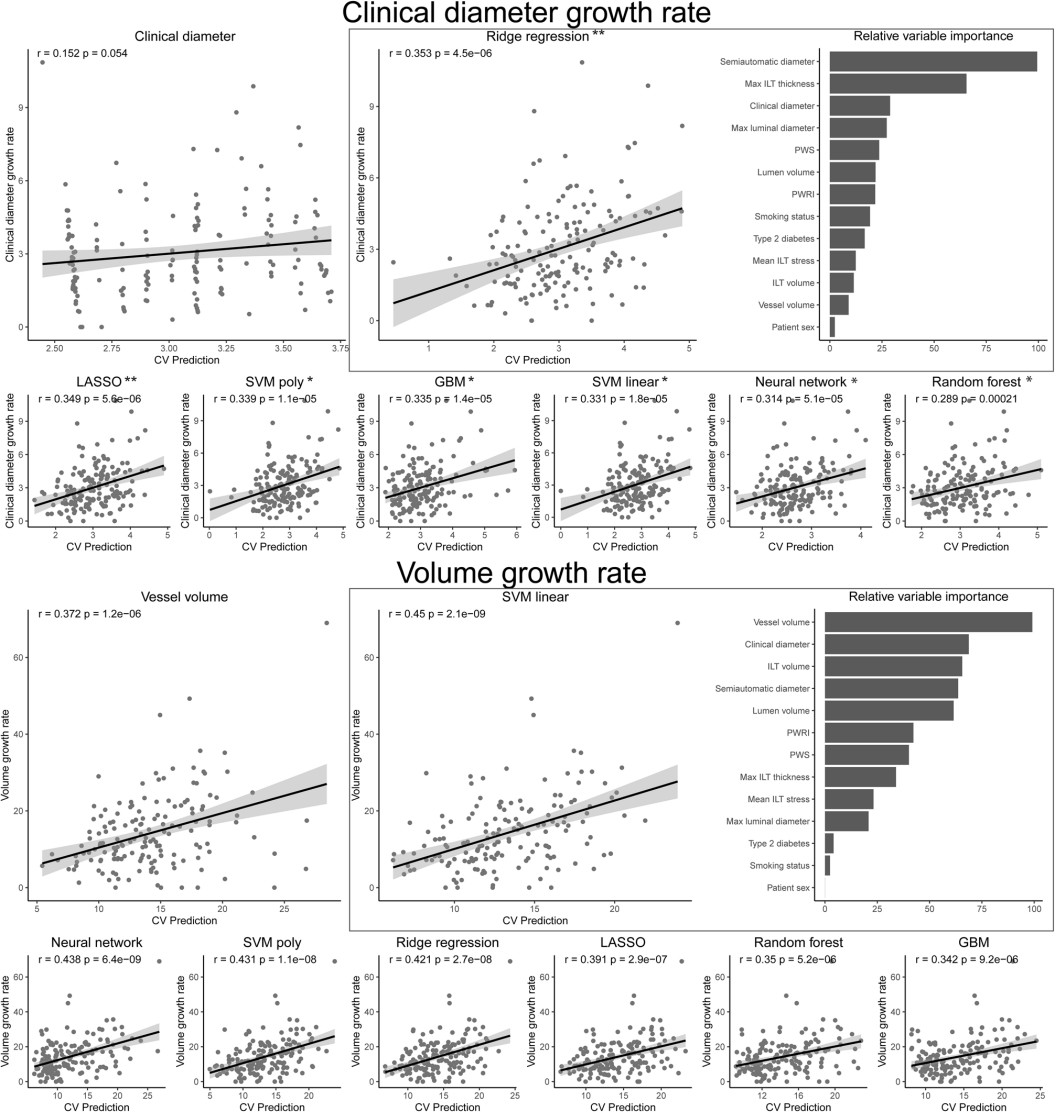
Prediction of aneurysm growth rate. Pearson correlation tests between the observed growth rates of clinical diameter or vessel volume and the cross-validated prediction. Linear regression of clinical diameter or vessel volume served as references. Models with significantly stronger correlation coefficients between prediction and observation, compared with the reference, were marked with stars. Variable importance is shown for the best models. *Abbreviations*; CV: cross-validated. GBM: gradient boosting machines, ILT: intraluminal thrombus, LASSO: least absolute shrinkage and selection operator, PWRI: peak wall rupture index, PWS: peak wall stress, SVM: support vector machine with linear or radial basis function kernel. Notations: *: *p* < 0.05, **: *p* < 0.01.

Growth rates were examined in patients with two CTAs performed within 8 months to 8 years intervals (n = 161). Geometric-biomechanical models improved the prediction of clinical diameter growth rate compared with the clinical diameter at baseline and the correlations between predicted and observed values were significantly different (r = 0.35, *p* < 0.001 for the best-performing model and r = 0.17, *p* = 0.033 for clinical diameter reference, correlation comparison *p* = 0.0061, Fig. 3). Analysis of variable importance revealed that the semiautomatic diameter was the most influential (Fig. 3) and the clinical diameter growth rate could be predicted with similar precision using this measurement alone as for the geometric-biomechanical model (not shown). The prediction of volume growth rate was nominally improved by these models compared with the baseline volume reference and there was a trend of difference between the correlation coefficients (r = 0.45, *p* < 0.001 for the best-performing model vs r = 0.37, *p* < 0.001 for the reference, correlation comparison *p* = 0.081, Fig. 3).

In a post-hoc analysis, all potential combination of variables (i.e. 2^13^ combinations) were tested in tenfold cross-validated multiple regression repeated 10 times. To predict clinical diameter growth rate, using clinical and semiautomatic diameter, max ILT thickness, lumen volume, ILT volume, smoking and type 2 diabetes were the most successful (r = 0.43 between predicted and true growth rate) but similar performance was obtained using only semiautomatic and clinical diameters (r = 0.41). To predict volume growth rate, the combination of clinical diameter, max ILT thickness, PWS and smoking were the most successful (r = 0.45) but with similar performance obtained using only clinical diameter and vessel volume (r = 0.43).

### Ruptured or symptomatic aneurysm

**Figure 4:**
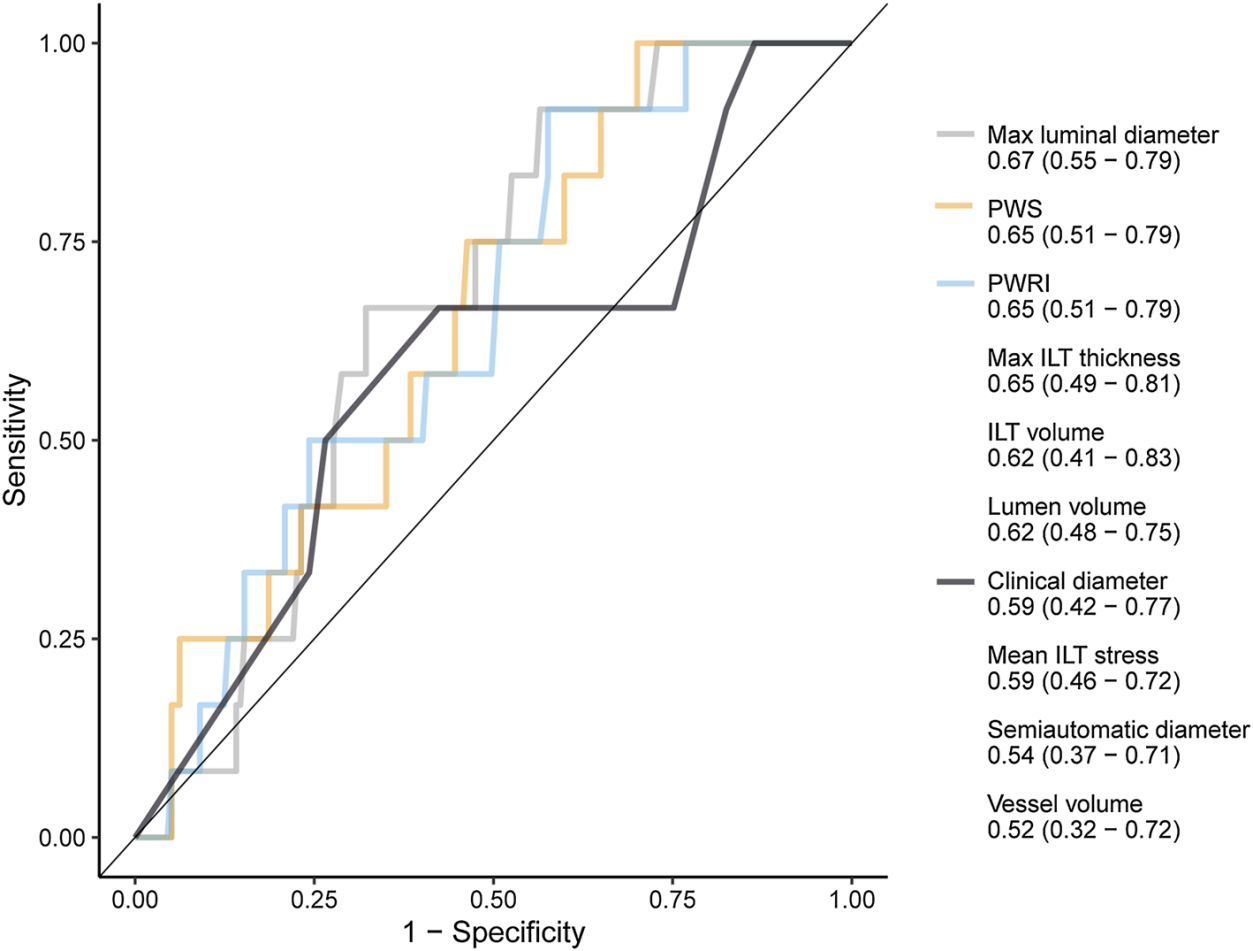
Receiver operating characteristic curves for the prediction of future rupture or symptomatic aneurysm. The area under curve (95% confidence interval) is presented below each variable. Only significant curves and the clinical diameter reference are displayed. *Abbreviations*; ILT: intraluminal thrombus, PWRI: peak wall rupture index, PWS: peak wall stress, WS: wall stress.

Five (2.6%) of the included patients later suffered from rupture and 7 (3.7%) developed a symptomatic AAA. The limited number of cases did not allow for prediction modeling or cross-validation. However, the geometric and biomechanical variables were evaluated with univariate ROC analysis (Fig. 4). The only features with AUCs significantly larger than 0.5 (i.e. better than chance) were luminal diameter, PWRI and PWS with AUCs (95% confidence intervals) of 0.67 (0.54–0.79), 0.65 (0.51–0.79) and 0.65 (0.51–0.79). The differences between AUCs were not significant. The time from index CT to rupture or symptomatic AAA was long, with median 4.8 (min: 1.7, max 9.7) years.

## Discussion

Prediction of the natural history of AAAs on the individual level remains a challenge, as does identification of the few patients who rupture during surveillance. The approach of the present study was to integrate a number of geometric and biomechanical variables derived from 3D modeling and FEA by use of machine learning in order to enhance the accuracy of such predictions. The predictions of growth rates and reaching surgical threshold within four years were enhanced by including all geometric and biomechanical variables in the prediction models, mainly due to the combination of clinical and semiautomatically measured external aneurysm diameters. In contrast, rupture or symptomatic AAA could only be significantly predicted with lumen diameter, PWRI and PWS, but not by external diameters.

The ability to accurately determine which patient with AAA that will require surgery within a certain time frame, and which patient will not, would improve risk/benefit analyses and simplify surveillance protocols. The geometric-biomechanical models used in the present study were separately trained to predict two outcomes; the aneurysm reaching the surgical threshold within four years as well as its growth rate measured in diameter or volume. Compared with the clinical diameter, it was possible to improve predictions of which patients would require surgery within four years. With the LASSO model, it was possible to reach 100% sensitivity with 21% specificity, and even 26% specificity when only patients with complete follow-up were included. It was not possible to reach 100% sensitivity with any specificity when only the clinical diameter and patient sex were considered. Predictions of the clinical diameter growth rate and, nominally, the volume growth rate were also improved by these models. When examining variable importance, i.e. how much each variable influences each model, it was clear that the semiautomatic and clinical diameters were highly influential. When only these two measurements were used together as predictors of reaching the threshold for surgery, or only the semiautomatic diameter to predict clinical diameter growth, the same improvements were achieved as with the more complex geometric-biomechanical models. Our interpretation is that by measuring the diameter automatically from a 3D model in addition to the ‘manual’ measurement performed in the clinic, an increased precision is attained. Being able to robustly predict who will reach a threshold for surgery, including the occurrence of rupture or symptomatic AAA, with 100% sensitivity and 21–26% specificity allows the safe scheduling of the next diameter measurement four years into the future for one fifth to one fourth of patients with small AAAs. These results speak to a potential value of conducting CTA at an early stage during surveillance in addition to the standard pre-operative examination.

A small share of the included patients suffered from rupture or developed a symptomatic AAA (6.3%). As patients were included based on a CTA examination of a small, asymptomatic AAA, irrespective of future developments, these numbers are representative of those seen at a contemporary vascular surgery department. The only variables that could predict these events were lumen diameter, PWS and PWRI whereas clinical and semiautomatic diameters could not. These results are in line with previous literature. Specifically, both PWS and PWRI (or equivalents) have been proposed as superior predictors of rupture compared with diameter^27–31,33,35,40,49–52^, whereas two studies did not observe a significant difference^32,53^. The size of the lumen as a marker of rupture risk has also recently been described by our group and others^54–56^. While yielding the only significant AUCs, the precision of PWRI, PWS and lumen diameter to predict rupture or symptoms in the present data was not excellent, with AUCs of 0.65 to 0.67. Importantly, the event occurred long after the initial CTA images, with an interval of between 1.7 and 9.7 years, and increased precision of PWRI to predict rupture in the near-term rather than long-term has recently been observed^33^.

Radiological, biomechanical and molecular markers of AAA growth rate have been studied previously. Geometric variables studied include the aneurysm volume, which has been suggested by our group and others as a more sensitive and easily predicted measurement of AAA growth^14,17^, as well as the shape and size of the ILT^5,20,21^. Moreover, some studies have examined the ability of biomechanical estimates to predict the growth rates of AAAs. Examples of approaches used previously are PWS in a model with local wall thickness estimated from CT images by a custom algorithm^34^, lumen volume and the computational fluid dynamics-derived wall shear stress in logistic regression^57^, and a measurement similar to PWRI to predict the combined event of elective repair or rupture^35^. Functional imaging strategies to predict AAA growth have relied on fluorodeoxyglucose (^18^F-FDG)^23,58^ and Fluorine-18–sodium fluoride (^18^F-NaF)^25^ positron emission tomography CT and ultrasmall superparamagnetic particles of iron oxide (USPIO) on magnetic resonance imaging^59^. Finally, a number of circulating biomarkers have been described^26^. While these studies are intriguing and the biomarkers are promising, most resulting decision rules have yet to be successfully compared with the clinically available maximal diameter in a validation set. Further, most studies have focused on single markers, and few have used machine learning approaches^60,61^. Integrating several of these markers into a machine learning framework could be fruitful going forward.

Some limitations of the current study merit consideration. While inclusion was performed consecutively based on two search strategies and the baseline CTAs analyzed were of a pre-event AAA, the retrospective nature of this study resulted in different imaging intervals, which were adjusted for by non-linear growth equations, and the incomplete follow-up of some patients. The number of ruptures and symptomatic AAAs was too small to allow the same rigorous evaluation as the growth and threshold for surgery data. Further, as only a small share of causes of deaths are verified by autopsy, some instances of lethal AAA rupture not diagnosed at a hospital may have been missed. Validation, strengths and limitations of the employed biomechanical methodology have been thoroughly reviewed in previous literature^28,37,62,63^. The method is fast, easy to use and computes PWS and PWRI (as well as mean ILT stress) but not shear stresses which requires computationally demanding and time-consuming blood flow simulations. While consideration of blood flow in addition to blood pressure seems to have negligible effects on PWS estimations^64^ and thus not deemed crucial for rupture risk estimations, our group and others have previously noted an association between shear stresses and growth^57,65^.

## Conclusions

Geometric and biomechanical variables integrated by machine learning approaches improved prognostication of small AAAs. Increased precision of external diameter measurements by use of 3D modeling improved the accuracy of prediction of future surgery and growth rate in AAAs, whereas other geometric and biomechanical indices did not result in additional improvement. In contrast, lumen diameter, PWS and PWRI, but not external diameters, predicted future rupture or symptomatic AAA. Predicting growth rates and rupture in patients with small AAAs thus require different models. Further increase in precision than what was attained in the current data would be desirable. Investigations of additional features such as functional imaging, simulation of blood flow, strain mapping and circulating molecular biomarkers are warranted.

## Supplementary Information

Supplementary Figures.
