# Supplementary material for: Geometric and biomechanical modeling aided by machine learning improves the prediction of growth and rupture of small abdominal aortic aneurysms
Source: Sci Rep. 2021 Sep 10;11:18040. doi: 10.1038/s41598-021-96512-3 (PMC8433325; doi:10.1038/s41598-021-96512-3)
Supplement: Supplementary file 1 — Supplementary Figures. [file 41598_2021_96512_MOESM1_ESM.pdf]

SUPPLEMENTARY INFORMATION

Geometric and Biomechanical Prediction Modeling of Growth and Outcome of Small Abdominal Aortic Aneurysms Using Machine Learning.

Moritz Lindquist Liljeqvist MD PhD*[1,3], Marko Bogdanovic MD[1], Antti Siika MD[1], T. Christian Gasser PhD[2], Rebecka Hultgren MD PhD[1,3], Joy Roy, MD PhD[1,3]

1. Department of Molecular Medicine and Surgery, Karolinska Institutet, Stockholm, Sweden.
2. Department of Engineering Mechanics, Royal Institute of Technology, Stockholm, Sweden.
3. Department of Vascular Surgery, Karolinska University Hospital, Stockholm, Sweden.

*Corresponding author

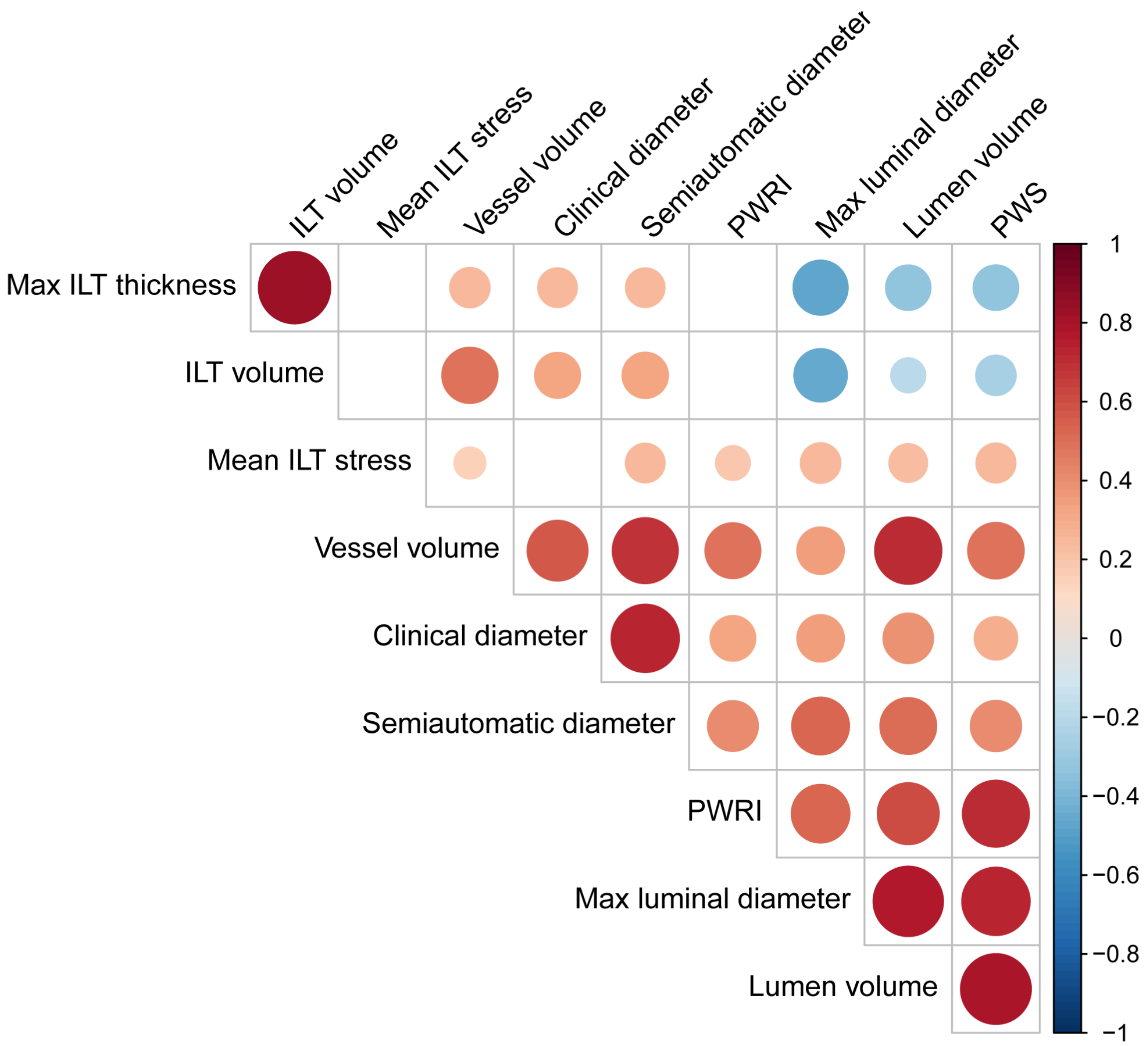

Figure S1: Correlations between geometric and biomechanical output variables. Color and size of the circles corresponds to the Spearman correlation strength. Abbreviations; GR: growth rate, ILT: intraluminal thrombus, PWRI: peak wall rupture index, PWS: peak wall stress.

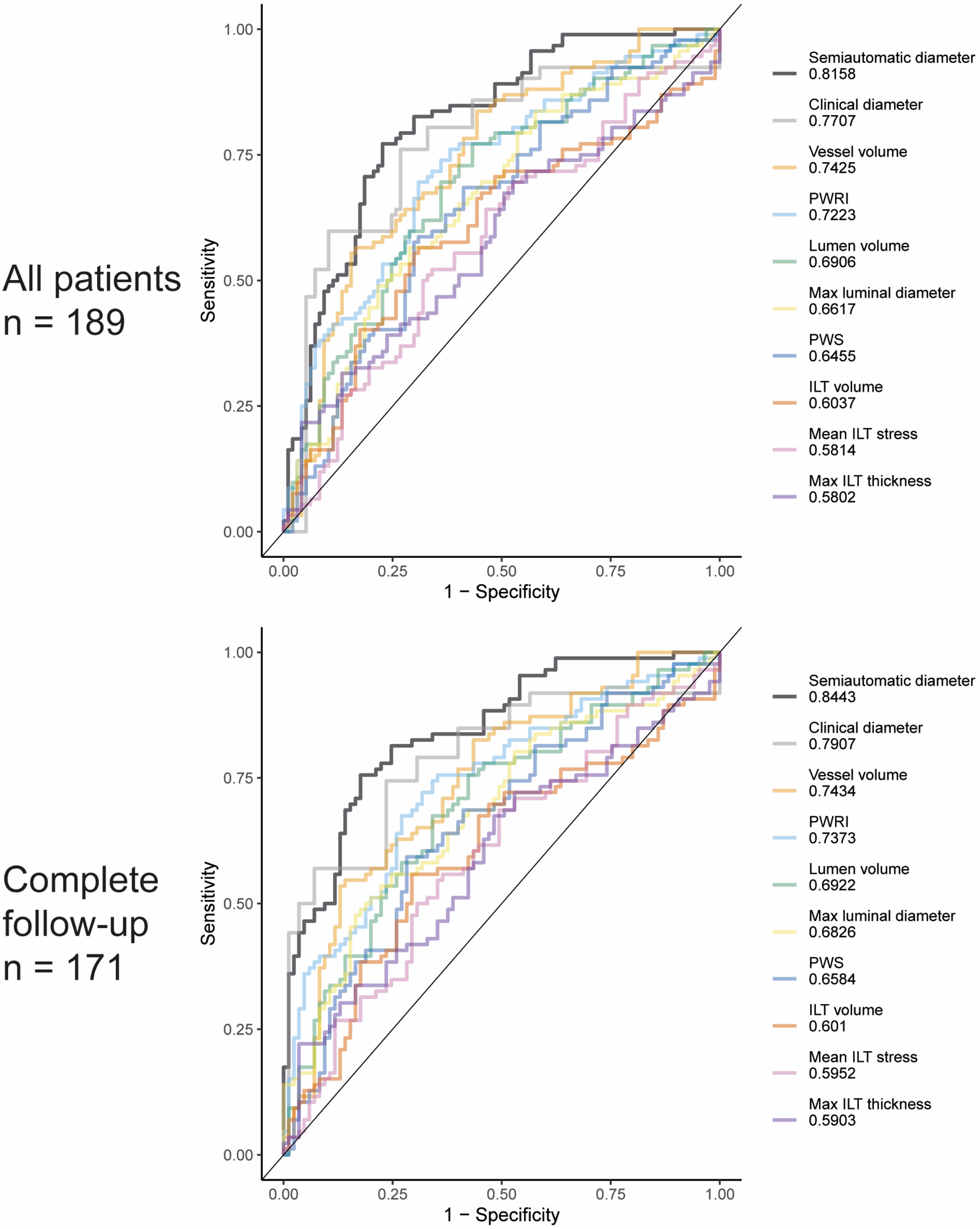

Figure S2: Prediction of reaching surgical threshold within four years by simple logistic regression. Predictions by simple logistic regression based on all included variables performed on all patients (upper figure) and only those with complete follow-up (bottom figure). The area under curve (AUC) is presented below each variable.

Abbreviations; ILT: intraluminal thrombus, PWRI: peak wall rupture index, PWS: peak wall stress.
